# Optimization of Contrast-to-Tissue Ratio by Adaptation of Transmitted Ternary Signal in Ultrasound Pulse Inversion Imaging

**DOI:** 10.1155/2013/297463

**Published:** 2013-03-20

**Authors:** Sébastien Ménigot, Jean-Marc Girault

**Affiliations:** ^1^IUT Ville d'Avray, Université Paris Ouest Nanterre La Défense, 92 410 Ville d'Avray, France; ^2^UMR-S930, Université François-Rabelais de Tours, 37032 Tours, France; ^3^U 930, Inserm, 37032 Tours, France

## Abstract

Ultrasound contrast imaging has provided more accurate medical diagnoses thanks to the development of innovating modalities like the pulse inversion imaging. However, this latter modality that improves the contrast-to-tissue ratio (CTR) is not optimal, since the frequency is manually chosen jointly with the probe. However, an optimal choice of this command is possible, but it requires precise information about the transducer and the medium which can be experimentally difficult to obtain, even inaccessible. It turns out that the optimization can become more complex by taking into account the kind of generators, since the generators of electrical signals in a conventional ultrasound scanner can be unipolar, bipolar, or tripolar. Our aim was to seek the ternary command which maximized the CTR. By combining a genetic algorithm and a closed loop, the system automatically proposed the optimal ternary command. In simulation, the gain compared with the usual ternary signal could reach about 3.9 dB. Another interesting finding was that, in contrast to what is generally accepted, the optimal command was not a fixed-frequency signal but had harmonic components.

## 1. Introduction

Intravenous injection of ultrasound contrast agents containing microbubbles has revolutionized medical ultrasound imaging in the past twenty years by making possible extraction of physiological and pathological information [1]. Subsequently, the contrast between the tissue perfused by the microbubbles and the nonperfused tissue, that is, contrast-to-tissue ratio (CTR), has been improved by taking into account the nonlinear behaviour of microbubbles, as in the second harmonic imaging [2], subharmonic imaging [3], superharmonic imaging [4], and attenuation correction [5].

However, the effects of the propagation of the ultrasound wave have limited these improvements since the tissue can generate nonlinearities, thereby reducing the CTR. Furthermore, since a good separation of the harmonic components requires a limited pulse bandwidth [6], the axial resolution has been limited. To overcome this drawback, certain discrete encoding techniques such as pulse inversion imaging [7], power modulation [8], contrast pulse sequencing [9], and pulse subtraction [10] have been developed to ensure a good axial resolution while increasing the CTR. Finally, to solve the trade-off between resolution and penetration, other imaging methods such as harmonic chirp imaging [11] have extended this principle for continuous encoding.

However, whatever the imaging system used in clinical practice, the fact remains that the excitation settings are manufacturer and user dependent. From our point of view, these settings are not optimal, since they must take into account the explored medium. For adjusting these settings to any examination, it is necessary to correctly adjust this excitation. To do so, the optimal command framework in which the problem takes place is presented. Thus, the problem can be written in such a way that the optimal command *x* ^⋆^(*n*) of the ultrasound imaging system provides the best CTR:

(1) $$\begin{array}{c} x^{⋆}\left(n\right)=\underset{x\left(n\right)}{\text{argmax}}\left(\text{CTR}\left(x\left(n\right)\right)\right), \end{array}$$

where *x*(*n*) is the signal transmitted and *n* is the discrete time.

Some solutions have been already proposed to solve (1) by either minimizing the tissue backscattering or maximizing the microbubble backscattering. In this context, two interesting approaches have been proposed. On the one hand, time reversal imaging only makes it possible to reduce the nonlinearities of the tissue backscattering [12]. Unfortunately, the formalism is linear and cannot take into account the microbubble nonlinearities to maximize them. On the other hand, an analytic solution has been proposed for the microbubble backscattering [13]. However, this theoretical solution requires the knowledge of all physical properties about the microbubble, the surrounding medium, and the transducer. This *a priori* information which can be accessible with difficulty, even completely inaccessible, led this analytical solution to be inapplicable in practice.

To overcome these limitations, a novel method has recently been proposed. This approach solves (1) by transforming a shape optimization into a suboptimal parametric optimization [14]. In this latter work, the parameter to be optimized was the transmit frequency. Thus, the optimal frequency was the transmit frequency which optimized the cost-function CTR. The computation of this optimal frequency was obtained automatically by using a simple algorithm based on the gradient. Although this method is simple, it lays the groundwork of the optimal command. Unfortunately, this approach is not completely satisfactory since the initial fixed waveform may not be suitable [15]. Furthermore, this approach does not take into account the specific features of electrical signal generators in conventional ultrasound scanners. For these ultrasound scanners, transmitters are usually unipolar (voltage impulse V), bipolar (voltage −V or V), or tripolar (voltage −V, 0, or V) generators, because their electronic conception is easier. Recently, the nature of the generator has been taken into account in order to improve the signal-to-noise ratio [16] and microbubble detection [17] by combining a binary waveform and an advanced imaging approach. However, no input optimization process has yet been proposed to find the optimal command.

Finally, no method has been proposed to date to overcome this problem satisfactorily and optimally by taking into account the tripolar transmitter constraint. Since (i) one of the most commonly used ultrasound imaging approaches is pulse inversion imaging and (ii) a conventional transmitter is a tripolar generator, the aim of this study was to automatically determine the optimal ternary command for the ultrasound pulse inversion imaging system to provide the best CTR:

(2) $$\begin{array}{c} x^{⋆}\left(n\right)=\underset{x\left(n\right)=\left\{-1,0,1\right\},\forall n}{\text{argmax}}\left(\text{CTR}\left(x\left(n\right)\right)\right). \end{array}$$

We therefore modified the current system (including a tripolar ultrasound transmitter) by including feedback. To resolve the digital waveform optimization, we proposed using a genetic algorithm through simulations. The advantage of the method was that no *a priori* information was required in order to find the optimal ternary command.

## 2. Closed-Loop System

**Figure 1:**
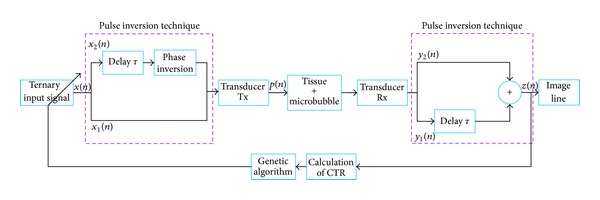
Block diagram of CTR optimization in pulse inversion imaging.

 The principle of pulse inversion imaging including feedback is described in Figure 1. For an individual solution at the iteration *k*, two ternary pulses, *x*_*k*,1_(*n*) and *x*_*k*,2_(*n*), with opposite phases were transmitted. The sum *z*_*k*_(*n*) of the two respective echoes, *y*_*k*,1_(*n*) and *y*_*k*,2_(*n*), formed a radiofrequency line *l*_*k*_. By taking into account the CTR_*k*_ estimated on this radiofrequency line *l*_*k*_, a new transmitted ternary signal *x*_*k*+1_(*n*) was proposed by the algorithm to optimize the CTR_*k*+1_.

### 2.1. Transmitted Ternary Signal

The ternary pulse signal *x*_*k*,*q*_(*n*) was digitally computed with MATLAB (MathWorks, Natick, MA, USA):

(3) $$\begin{array}{c} x_{k,q}\left(n\right)=\{\begin{array}{cc} A\cdot w_{k}\left(n\right) & \text{if}\;\;q=1,\\ -A\cdot w_{k}\left(n\right) & \;\text{if}\;\;q=2. \end{array} \end{array}$$

The ternary signal *w*_*k*_(*n*) was defined on a duration *T*, which corresponded to 100% of the fractional bandwidth of the transducer. It was thus constructed from *N*_*s*_ samples, where each sample could take the value −1, 0, or 1.

The amplitude of the driving pressure *A* was then adjusted so that the power of the pulse *x*_*k*,*p*_(*t*) was constant to *P*_*x*_ref__ for all ternary signals transmitted:

(4) $$\begin{array}{c} A=\sqrt{\frac{A_{0}^{2}\cdot P_{x_{\text{ref}}}}{P_{w}}}, \end{array}$$

where the power *P*_*x*_ref__ was calculated for a signal *x*_ref_ which was the impulse response of the transducer with a driving pressure *A*_0_. The power *P*_*w*_ was the power of the signal *w*_*k*,*p*_. The power of the transmitted wave thus remained constant by adjusting the amplitude signal *A*. 

### 2.2. Cost Function

The aim was to maximize the contrast between the tissue perfused by the microbubbles and the nonperfused tissue by selecting the transmitted signal *x*(*n*). Since the usual contrast estimator in ultrasound contrast imaging is the CTR, the cost function was CTR_*k*_ computed from a line *z*_*k*_(*n*):

(5) $$\begin{array}{c} z_{k}\left(n\right)=y_{k,1}\left(n\right)+y_{k,2}\left(n\right), \end{array}$$

where *y*_*k*,*p*_(*n*) is the echo of the transmitted pulse *x*_*k*,*p*_(*n*). Thus, the CTR_*k*_ is defined as the ratio of the power *P*_*b*,*k*_ backscattered by the area of the perfused medium to the power *P*_*t*,*k*_ backscattered by the area of the nonperfused medium [18] as follows:

(6) $$\begin{array}{c} {\text{CTR}}_{k}=10\cdot \underset{10}{\log}\left(\frac{P_{b,k}}{P_{t,k}}\right). \end{array}$$

These powers were computed from the lines *z*_*k*_(*n*) of the pulse inversion image. Note that the areas were delineated manually before the optimization process, but a segmentation step could be implemented to help the delineate process.

### 2.3. Genetic Algorithm

The seeking of the optimal excitation *x* ^⋆^(*n*) consisted in (i) transmitting ternary stochastic signals *w*_*k*_(*n*) through the medium and in (ii) selecting the optimal ternary signal which maximized the cost function. However, since this latter step required a large number of ternary stochastic signals, to reduce the computational time, we proposed using a metaheuristic. This metaheuristic based on the principle of biological reproduction [19] is a genetic algorithm. It found the optimum by setting a chromosome [20], that is, a vector composed of *N*_*s*_ samples of the ternary signal *w*_*k*_(*n*).

 The ternary genetic algorithm was based on a binary genetic algorithm [21]. In our case, at each iteration *k*, a generation *k* with *M* ternary individual solutions (sample vectors) was tested, where the probability of the sample value was uniform between −1, 0, or 1. As proposed in [21], the number *M* of individual solutions per generation was 12.

 For the next generation *k* + 1, the selection operator only conserved the *M*/2 best individual solutions which maximized the CTR. These vectors became pairs and mates. The best parent was then mixed with one of the *M*/2 − 1 remaining parents by the crossover operator. The offspring was constituted of part of the first parent samples until the crossover point and part of the second parent samples from the crossover point. Note that the crossover point was randomly selected between the first and the last sample. An offspring of *M*/2 new individual solutions thus contained the ternary signal of both parents.

Finally 40% of the samples were mutated so that the optimization was robust. The best individual solution was the optimal ternary command for the generation *k*. Note that a small population and a high mutation rate were chosen to solve the trade-off between robustness and the computation time due to sorting of each individual solution [21].

## 3. Simulation Model

 The simulation model was constructed on the pulse inversion imaging system (Figure 1). It was composed of different phases: transmission, 2D nonlinear propagation, nonlinear oscillations of microbubbles, and reception [14]. A pulse wave was propagated nonlinearly into an attenuating medium without microbubbles. This wave, composed of harmonic components, excited a microbubble in the vascular system. The nonlinear oscillations of this microbubble were backscattered and measured by the receiver.

### 3.1. Nonlinear Propagation in Tissue

A ternary signal *x*_*k*,1_(*n*) was generated digitally and filtered by the transfer function of a realistic transducer, centred at *f*_*c*_ = 4 MHz with a fractional bandwidth of 75% at −3 dB. The 2D nonlinear wave propagation into the medium was obtained by solving Anderson's model based on a pseudospectral derivative and a time-domain integration algorithm [22]. This solver required three grids: a grid of mean density of 928 kg · m^−3^, a grid of mean speed of sound of 1578 m · s^−1^, and a grid of *B*/*A* nonlinearity parameter of 6.7 [23]. The scatterers were generated randomly by weakly modifying the density grid of ±0,5 kg · m^−3^ and the speed grid of ±0,5 m · s^−1^. Note that an attenuation of 0.45 dB · MHz^−1.05^ · cm^−1^ was used. Finally, the signal backscattered by tissue was recorded, and the driving pressure at 15 mm was included into the microbubble model described below.

### 3.2. Microbubble

The simulated ultrasound contrast agent had the properties of encapsulated microbubbles used in clinical practice with a mean diameter of 2.5 *μ*m [24] and a resonance frequency of 2.6 MHz [25]. The acoustic response was computed for one microbubble from Marmottant's model [26] based on the Rayleigh-Plesset equation and the polytropic transformation. Finally, since the pressure was low in comparison with the transmitted pressure, the echo of the microbubble was deduced from the oscillation [27] without including nonlinear propagation. Note that in order to simulate the mean behaviour of a microbubble cloud, we hypothesized that the response of a cloud of *N*_*b*_ microbubbles was *N*_*b*_ times the response of a single microbubble with the mean properties. To simplify, the microbubble response was thus multiplied by *N*_*b*_ in order to simulate a mean nonlinear behaviour of a 1/2000 diluted microbubble cloud. Moreover, to be more realistic, the attenuation effects due to the high concentration of microbubbles were taken into account [28] for this dilution.

The echoes from tissue and microbubbles were added and filtered by the transfer function of the transducer to construct the first echo for the transmitted signal *x*_*k*,1_(*n*). The simulation process was repeated for the transmitted ternary pulse *x*_*k*,2_(*n*) to construct the second echo. Finally, the radiofrequency line *l*_*k*_ was constructed from *z*_*k*_(*n*) described by (5).

## 4. Results

The optimization process was applied to the previous simulation model. The driving pressure *A*_0_ was set at 400 kPa. The duration *T* of the ternary signal represented 100% of the fractional bandwidth of the transducer, that is, *T* = 0.3 *μ*s. According to the sampling rate required by the simulation model, there were 40 samples in 0.3 *μ*s; therefore, *N*_*s*_ = 40.

To demonstrate the efficacy of the new method, the results were compared to those of the two usual transmitted signals. To construct them, Gaussian-modulated sinusoidal pulses were digitalized to obtain a ternary signal. Their bandwidth represented 100% of the fractional bandwidth of the transducer, and their transmitted power was *P*_*x*_ref__. Their transmit frequencies were set at (i) two-thirds of the central frequency *f*_*c*_ of the transducer [29] (2/3*f*_*c*_ = 2.67 MHz) and at (ii) the optimal frequency *f*_opt_. Note that this optimal frequency *f*_opt_ enabled to maximize the cost function CTR as presented in [14].

Table 1 summarizes the CTR measured for the optimal ternary signal and the two usual ternary signals. By using frequency optimization, it was possible to achieve a suboptimal solution, better than the transmitted signal at the usual transmit frequency. However, the CTR was higher with the transmitted ternary signal. This CTR value could not be achieved with the usual ternary signal digitalized from a Gaussian-modulated sinusoidal pulse, although the transmit frequency was optimized.

**Figure 2:**
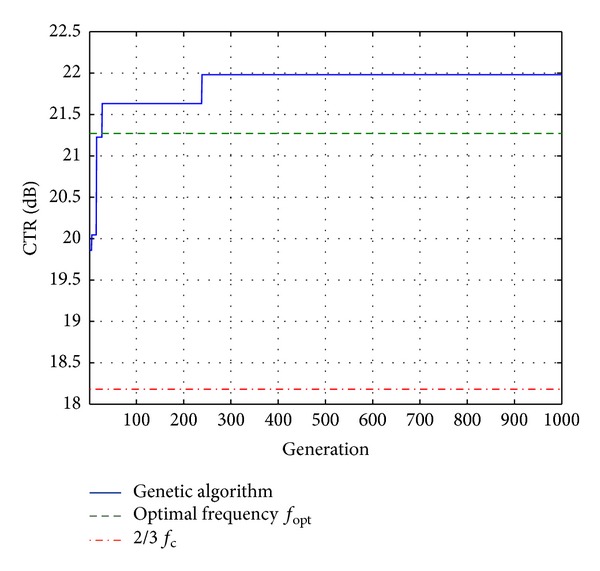
Simulation of automatic optimization of the contrast-to-tissue ratio (CTR) by a transmitted ternary signal. The optimization was compared to two ternary signals, where the transmit frequency was at the optimal frequency and at two-thirds of the central frequency of the transducer.

Figure 2 shows the best CTR as a function of generation *k*. As an illustration, this result was compared with the two usual ternary signals. After 239 generations, the CTR achieved an optimal value that was higher than the frequency setting cases. The gain reached 3.9 dB in comparison with the usual fixed-frequency transmitted signal and 0.8 dB in comparison with the transmitted signal at the optimal frequency *f*_opt_.

**Figure 3:**
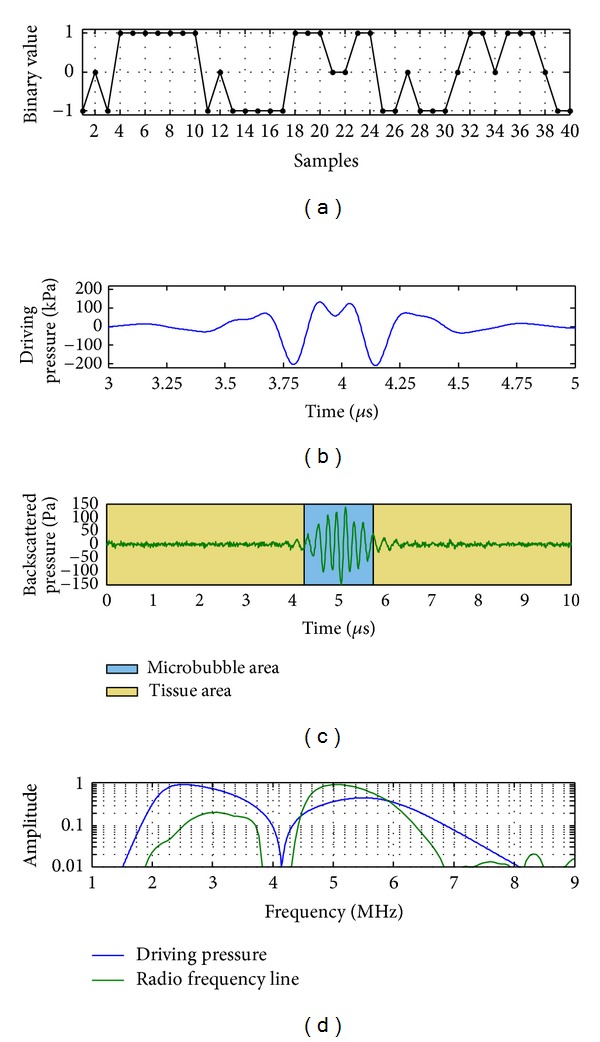
(a) Optimal transmitted ternary signal *x*_1_(*n*) obtained by genetic algorithm, (b) Signal *p*(*n*) at the transducer output (Figure 1), when *w*(*n*) was the optimal ternary signal, (c) the radiofrequency line, and (d) their spectra.

Figure 3(a) shows the optimal ternary command *w*_opt_(*n*). As an illustration, Figure 3(b) shows the signal *p*(*n*) at the transducer output (Figure 1) transmitted to the tissue when *w*(*n*) was the optimal ternary signal (Figure 3(a)), and the corresponding backscattered signal was shown in Figure 3(c). Their respective spectra were presented in Figure 3(d). Unlike a usual fixed-frequency transmitted signal, the optimal transmitted signal had nonlinear components. Note that the nonlinear components backscattered by the tissue and the microbubbles remained, because in pulse inversion imaging the linear component was suppressed. 

## 5. Discussion and Conclusions

 From results derived from Figure 2, the optimal command methods, presented in [14] and here, outperformed the nonoptimized reference method. Although these two methods are optimal command methods, they presented some significant differences. The first method [14] by imposing a waveform defined by a frequency parameter constitutes an optimal monoparametric solution, and the second method proposed here by imposing a ternary constraint on the waveform constituted an optimal multisample solution.

In this latter method, ternary sequences were automatically transmitted through a pulse inversion imaging system in order to optimize the CTR. This optimization was performed without taking into account *a priori* information about the medium or the transducer, except the fact that this method required a selection of two regions of interest (with and without microbubbles). The delineation of these regions of interest constituted both the strength of the method, since it enabled to define the CTR cost function and a drawback for fully perfused tissue for which no CTR computation is possible. Note that to partly overcome this problem, it can be recommended to change the organ section in a view to delineate a nonperfused area.

Nevertheless, by disregarding the latter drawback, the closed-loop system had the advantage to provide an optimal ternary command. By using this optimal ternary command, the CTR was higher than that with the usual ternary signals digitalized from Gaussian-modulated sinusoidal pulses at a fixed transmit frequency. This optimal setting proposed a filtered ternary wave composed of harmonic components transmitted to the medium being explored. While most researchers have focused on using a fixed-frequency transmitted signal, the better solution was to find a transmitted signal composed of harmonic components. These harmonic components present in the transmitted signal did not affect the CTR, because the pulse inversion properties ensured the extraction of nonlinearities generated only by the medium. This property may explain the compromise between maximizing microbubble backscattering and minimizing tissue backscattering. Furthermore, since the transducer bandwidth was not broad enough, the double frequency of the second harmonic component was not present in the backscattered signal. However, since the process reached the optimum without the presence of the double frequency of this component, the only presence of a linear interaction acting on the transmitted second harmonic component seemed to play a crucial role in the optimization process.

Finally, the last advantage which seemed to be important was that the method automatically adjusted the transmitted ternary signal for any nonlinear and attenuating medium to be explored. The reason of this benefit was that the cost function, exclusively computed from the mean power of the output system, was independent of the microbubble size distribution. Indeed, as the backscattered mean power corresponds to an average operating on the whole spectrum, thus its value is independent of the frequency distribution, whether the spectrum had a narrow bandwidth (same microbubble size) or a large bandwidth (polydisperse microbubble size). Thus, even if the assumptions of the simulation model were simplified, the process of the CTR optimization completely ignored the nature of different underlying processes as the multiple scattering or the microbubble speckle. The method can therefore be applied to any medium to be explored.

For future integration in an ultrasound imaging system, the time to achieve optimization is crucial. Firstly, the CTR computation from regions of interest (*L* × *L* size) in the image required 2(2*L* + 1)2 + 1 operations. Secondly, the genetic algorithm required 0.4(12*N*_*s*_) + 6 random selections per generation to achieve the optimum. Taking into account the computing power available for a personal computer, the two last operations must not slow down the optimization process. However, the number of generations to achieve the optimum may be a limiting factor. Since the frame rate can reach 2000 Hz in some ultrasound scanners, this limitation should be relative. We therefore estimated that the optimization should take less than 5 seconds.

To conclude, the method reported ensured optimal CTR by selecting the appropriate transmitted ternary signal. The method could be applied to ultrasound imaging without using programmable analogue transmitters in contrast to transmit frequency optimization. Manufacturers and clinicians would not themselves need to tune the transmitted signal. This new approach could open up optimal commands for ultrasound imaging. The next step will be to implement it in an ultrasound scanner. Moreover, the future approach should take into account the fact that the optimal transmitted signal must be composed of harmonic components. 

## Figures and Tables

**Table 1:** CTR measured if the signal transmitted is (i) a ternary signal at two-thirds of the central frequency *f*_*c*_ of the transducer, (ii) a ternary signal at the optimal frequency *f*_opt_, or (iii) the optimal ternary command.

	2/3*f*_*c*_ = 2.67 MHz	*f*_opt_ = 1.9 MHz	Optimal ternary signal
CTR (dB)	18.1	21.2	22
